# Tracing the pathogenic PLN p.(Arg14del) variant across the globe; more than just a local curiosity

**DOI:** 10.1007/s12265-026-10792-6

**Published:** 2026-06-25

**Authors:** Esmée van Drie, Freyja H. M. van Lint, Rob Zwart, Jie Wang, Yucheng Chen, Alex V. Postma, Martin G. Elferink, Joris J. M. van Steenbrugge, Paul A. van der Zwaag, Jan D. H. Jongbloed, Dennis Dooijes, Myrthe Y. C. van der Heide, Arjan C. Houweling, Kristina H. Haugaa, Ida Skrinde Leren, Anna Kostareva, Hendrik Milting, Thuy Vy Nguyen, Thuy Duong Ho Huynh, Philippe Chevalier, Antoine Delinière, Juan R. Gimeno-Blanes, María Sabater-Molina, Roberto Barriales-Villa, Andrea Mazzanti, Mirella Memmi, Yuki Kuramoto, Tomoka Tabata, Arthur A. M. Wilde, Karin Y. van Spaendonck-Zwarts, J. Peter van Tintelen

**Affiliations:** 1 Department of Genetics, University Medical Center Utrecht, Lundlaan 6, 3584 EA Utrecht, the Netherlands; 2 Netherlands Heart Institute, Moreelsepark 1, 3511 EP Utrecht, the Netherlands; 3 Department of Genetics, Radboud University Medical Center, Geert Grooteplein Zuid 10, 6525 GA Nijmegen, the Netherlands; 4 Department of Human Genetics, Amsterdam UMC, Amsterdam, the Netherlands; 5 Department of Cardiology, West China Hospital, Sichuan University, Guoxue Alley No. 37, Chengdu, 610041 Sichuan China; 6 Department of Genetics, University of Groningen, University Medical Center Groningen, Hanzeplein 1, 9713 GZ Groningen, Netherlands; 7 Amsterdam UMC Location AMC, Department of Cardiology, University of Amsterdam, Meibergdreef 9, Amsterdam, the Netherlands; 8 Amsterdam Cardiovascular Sciences, Heart Failure and Arrhythmias, Amsterdam, the Netherlands; 9 ProCardio Center for Research Based Innovation, Department of Cardiology, Oslo University Hospital, Rikshospitalet, Sognsvannsveien 20, 0372 Oslo, Norway; 10 University of Oslo, Oslo, Norway; 11 Karolinska Institutet (KI), Solna, Sweden; 12 Heart and Diabetes Center NRW, Bad Oeynhausen, Germany; 13 Department of Genetics, Faculty of Biology and Biotechnology, University of Science, Vietnam National University, Ho Chi Minh City, Vietnam; 14 Research Center for Genetics and Reproductive Health, School of Medicine, Vietnam National University, Ho Chi Minh City, Vietnam; 15 Rhythmology Unit, Hospices Civils de Lyon, University of Lyon, Lyon, France; 16 Department of Cardiology, Virgen de Arrixaca Hospital, Ctra Murcia-Cartagena, S/N, 30120 El Palmar, Murcia, Spain; 17 Inherited Cardiovascular Diseases Unit, Hospital Universitario A Coruña, INIBIC/CIBERCV, As Xubias 84, 15006 A Coruña, Spain; 18 Department of Molecular Medicine, University of Pavia, ICS Maugeri, Molecular Cardiology, 27100 Pavia, Italy; 19 Department of Cardiovascular Medicine, Osaka University Graduate School of Medicine, Suita, Japan

**Keywords:** PLN, Cardiomyopathy, Clinical variability, Global prevalence, Haplotype, Founder

## Abstract

Studying the global distribution of the pathogenic variant c.40_42delAGA;p.(Arg14del) in the phospholamban (*PLN)* gene is highly important for raising awareness among healthcare providers and may help uncover factors contributing to variability in the development of associated cardiomyopathy phenotypes. PLN p.(Arg14del)-positive individuals were identified through a PubMed literature search, our clinical and research networks, and ClinVar. Additionally, population prevalences were determined using publicly available genetic databases. Furthermore, haplotype analysis was conducted using haplotype markers or whole genome sequencing data to assess whether newly identified cases across different continents share common ancestry. The PLN p.(Arg14del) variant was identified in 21 countries across four continents. Haplotype marker analysis suggest that most analyzed individuals, except those from Greece, shared at least part of a common haplotype. The PLN p.(Arg14del) variant is present in at least 2000 carriers globally. While the majority share at least part of a common haplotype, suggesting a common founder, data suggest an independent mutational event in Greek patients.

**Graphical Abstract:**

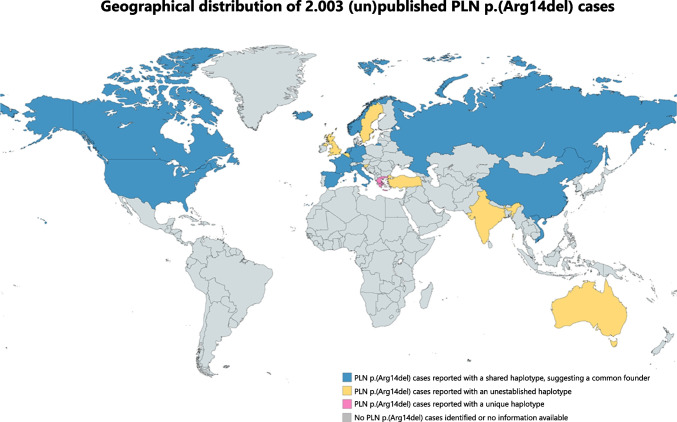

**Supplementary Information:**

The online version contains supplementary material available at 10.1007/s12265-026-10792-6.

## Introduction

Founder variants are not uncommon in inherited cardiac diseases, including cardiomyopathies [1–4]. Recurring genetic variants can be found in seemingly unrelated families that may, however, originate from common, often ancient ancestors. A recent study showed that recurring variants in arrhythmogenic right ventricular cardiomyopathy (ARVC), a major subtype of cardiomyopathy with unfavorable prognosis, are usually inherited and rarely occur de novo [3]. Founder effects are typically observed in geographically or culturally isolated areas [5]. However, this does not appear to be the case for the *PLN* c.40_42delAGA; p.(Arg14del) pathogenic variant which has been identified in many countries worldwide. In addition to ARVC, PLN p.(Arg14del) can also present as biventricular or dilated cardiomyopathy [6]. The variant was initially reported in a large Greek family in 2006[7], followed by studies from the U.S.A. [8], Germany [9], the Netherlands [10], Canada [11], and Spain [12]. Except for the Greek patients, carriers from these countries share a common haplotype surrounding PLN p.(Arg14del), suggesting a shared ancestral origin [10, 12]. More recently, this variant has also been described in patients from Belgium [13], China [14–16], France [17], Iceland [18], Italy [19], Japan [20], Norway [21], and Vietnam [22]. Most patients develop an arrhythmic or heart failure-related phenotype from late adolescence to older age, with an age-dependent penetrance of 43–70% by 70 years of age [23]. The factors underlying its clinical variability, as well as its age-dependent and even incomplete penetrance remain largely unknown [24–26]. However, recent findings suggest that the presence of additional rare genetic variants in a cardiogenetic-related disease gene may contribute to disease severity in PLN p.(Arg14del)-associated cardiomyopathy [27]. Additionally, current treatment regimens are often ineffective, with suboptimal response to guideline-directed heart failure therapy [28] and substantial risk of sudden cardiac death.

Investigating the global prevalence and haplotypes of PLN p.(Arg14del) may provide insight into its origin and global distribution. Moreover, examining cohorts from different populations may provide insights into factors contributing to its clinical variability. Furthermore, these cohorts may serve as external validation cohorts for current risk prediction models of PLN p.(Arg14del) carriers [29, 30]. Ultimately, this will have implications for optimizing patient selection for treatment of PLN p.(Arg14del)-associated cardiomyopathy, including novel precision medicine initiatives [31], such as gene therapy.

Therefore, this study aims to evaluate the global prevalence of the PLN p.(Arg14del) variant and analyze the surrounding haplotypes to determine whether newly identified cases across different populations share a common ancestral origin.

## Methods

### Global distribution of PLN p.(Arg14del)

We examined the global prevalence and distribution of PLN p.(Arg14del) by:

1. Identifying published cases by searching the PubMed database (accessed June 2024) using search terms “phospholamban” or “PLN” AND “c.40_42delAGA” or “R14del” or “Arg14del”.
2. Consulting our clinical and research networks, as well as ClinVar [32], to collect and confirm additional (un)published cases.
3. Reviewing large, publicly available population databases, including gnomAD v4.1.0 [33], UK Biobank [34], 54KJPN [35], and ‘All of Us’ initiative [36] (accessed June 2024). Additionally, other population and local genetic databases, i.e. deCODE genetics [37], Lifelines [38, 39], Groningen Area Controls [40], PREVEND [10] and Amsterdam Area Controls [41] were reviewed. deCODE genetics has provided us with the national allele frequency of this specific variant.

### Haplotype analyses to investigate founder effects of PLN p.(Arg14del)

To assess a potential founder effect, haplotypes were generated and compared to previously published data and those derived from an extended Dutch PLN population selected from the Netherlands Arrhythmogenic Cardiomyopathy registry [42]. Isolated DNA derived from peripheral blood samples from these PLN p.(Arg14del) patients was collected for haplotype marker analyses. If no DNA sample was available for haplotype marker analyses, but whole genome sequencing (WGS) data were, haplotypes were derived from WGS data.

We evaluated seven genetic markers spanning a region of 4.5 Mb, surrounding PLN c.40_42delAGA to determine the haplotype, as previously described [10] (Supplementary Fig. 1 and Supplementary Table 1). The phase of alleles was established as described previously [3]. All haplotype marker analyses were performed at the Amsterdam University Medical Center, except for the analysis of the Chinese samples, which was performed locally (West China Hospital, Chengdu, Sichuan, China) according to the identical protocol. For visualization purposes, haplotypes were converted to a Newick format displayed as a phylogenetic tree using R package ggtree (v3.6.2) [43]. For those patients sharing a haplotype, its age was re-evaluated, using the method described by Machado et al. [44], which was also used to estimate the age of PLN p.(Arg14del) haplotypes in the Dutch population [40].

WGS data of 58 additional Dutch PLN patients (who were not included in haplotype marker analysis) was available. As calling of the aforementioned seven genetic markers was not available in WGS data, 23 highly frequent (allele frequency 30–70%) single nucleotide polymorphisms (SNPs) surrounding *PLN* c.40_42delAGA (range ± 500 kb; Supplementary Table 2) were selected, for which the allele frequency was low (< 5%) in control populations (in-house WGS datasets and gnomAD). Derived from the set of 58 Dutch PLN patients, at least 16 contiguous SNPs (out of 23) were shared. Therefore, we assumed the presence of a common haplotype similar to that observed in Dutch PLN patients, when a patient was heterozygous for *PLN* c.40_42delAGA and shared at least 16 contiguous SNPs. This method was validated using control data of in-house WGS samples. If none of the 23 SNPs were shared, the presence of a common haplotype could however not be fully excluded, as the haplotype could be smaller than the genomic interval between the first two SNPs surrounding PLN c.40_42delAGA (< 200 kb). In such cases, SNPs in this particular region were selected for further analyses (Supplementary Note).

The study conforms with the principles of the Declaration of Helsinki and all participants gave informed consent according to local protocols (University Medical Center Utrecht, protocol UCC-UNRAVEL #12–387).

## Results

### Global prevalence and distribution of PLN p.(Arg14del)

1. A PubMed search revealed 79 publications and identified patients from nine countries. See Table 1 for an overview of the published number of carriers per country.
2. Via our clinical and research networks, we have collected and confirmed additional (un)published cases from eleven additional countries: Australia, Belgium, Denmark, France, Iceland, India, Norway, Russia, Sweden, Turkey, and the United Kingdom (Table 1). In ClinVar[32], additional cases were reported from laboratories in India, Slovenia, and Qatar; these cases have not been confirmed by involved health care providers yet.
3. The allele frequency of PLN p.(Arg14del) was available in Gnomad v4.1.0, deCODE genetics, Lifelines, Groningen Area Controls, PREVEND, and Amsterdam Area Controls and ranged between 1/131,061 and 1/473 (Table 1). The PLN p.(Arg14del) variant was absent in other publicly available databases, including the UK biobank, 54KJPN, and All of Us. However, inframe deletions in the *PLN* gene were probably not annotated in the underlying data.

**Table 1:** Occurrence of PLN p.(Arg14del) variant carriers based on published cases, unpublished cases, and population-based cohorts

Continent	Country	Published carriers (*N* =)	Unpublished carriers (*N* =)	Remarks	Population cohort frequency	
Europe	Belgium	1 [13]	10			
	Denmark		1	Norwegian origin		
	France	1 [17]	6	2 three-generation-families (1 family of Dutch origin and 1 family from central France		
	Germany	7 [9]	1			
	Greece	14 [7]				
	Iceland	42 [18, 45]	12	Total of 54 carriers belonging to a seven-generation family with common ancestors born in the late eighteenth century (around 1790)	deCODE genetics	1/3,200
	Italy	1 [19]		Paternal great-grandmother was of Dutch origin		
	Netherlands	933 [46]	379		Lifelines [39]	1/~ 500
					Groningen area controls [40]	1/473
					PREVEND [10]	1/~ 1,400
					Amsterdam area controls [41]	1/~ 500
	Norway	129 [21, 45]	151			
	Russia		1			
	Spain	20 [12, 29]	17			
	Sweden		1			
	Turkey		1			
	United Kingdom		1	Dutch origin		
	Total	1148	581		GnomAD	
					Non-Finnish	1/131,062
					Finnish	0
Asia	China	5 [14]^*^				
		5 [15]				
		8 [16]^*^				
	India		1			
	Japan	5 [20]				
	Vietnam	2 [22]				
	Total	25	1		GnomAD	
					East Asian	1/44,883
					South Asian	0
North America	Canada	34 [11, 45]				
	U.S.A	2 [8]	198			
		1 [47]				
		1 [48]				
		1 [49]				
	Total	39	198		GnomAD	
					Admixed American	0
					African American	0
Australia	Australia		11	2 families; 1 family linked to a Dutch relative		
	Total		11			

*Republication of the same individuals could not be excluded

**Fig. 1:**
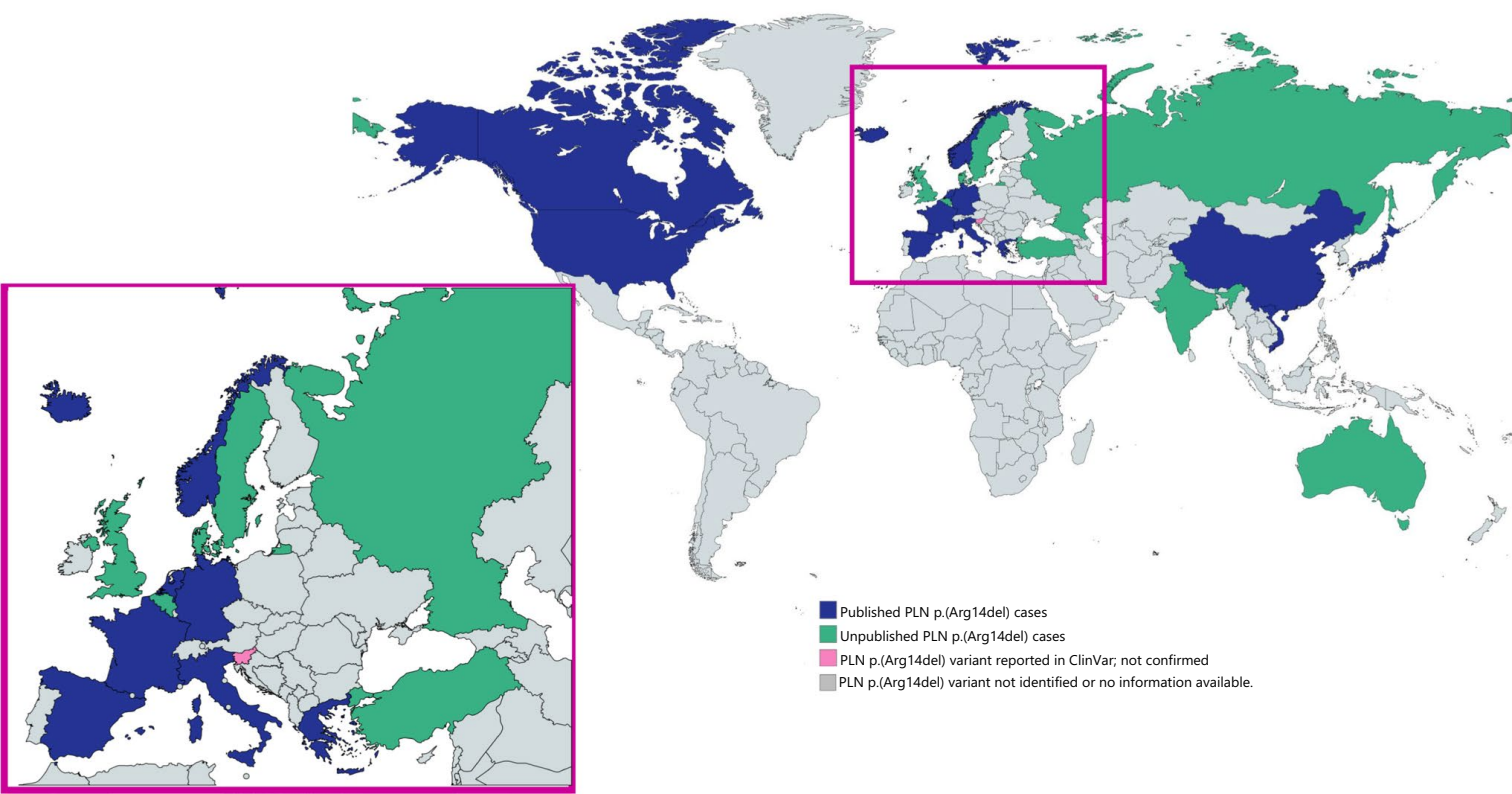
Global spread of *PLN* p.(Arg14del) variant. The geographical map provides an overview of countries with previously published cases (blue), and newly identified (for the purpose of this study) yet unpublished cases (green). In countries marked pink, *PLN* p.(Arg14del) has been reported in ClinVar, but has not yet been confirmed

In Fig. 1, a geographical map provides an overview of the globally identified PLN p.(Arg14del) cases in 21 countries across four continents.

### Founder effects and independent occurrences of the variant

For haplotype analyses we included patients with PLN p.(Arg14del) from China (*n* = 4) [15], France (*n* = 2) [17], Iceland (*n* = 20) [18], Italy (*n* = 1) [19], Norway (*n* = 2), Russia (*n* = 1), Vietnam (*n* = 2) [22] and from a region in the Northwest of Spain (*n* = 1; distinct from where the initial published Spanish patients reside [12]).

**Fig. 2:**
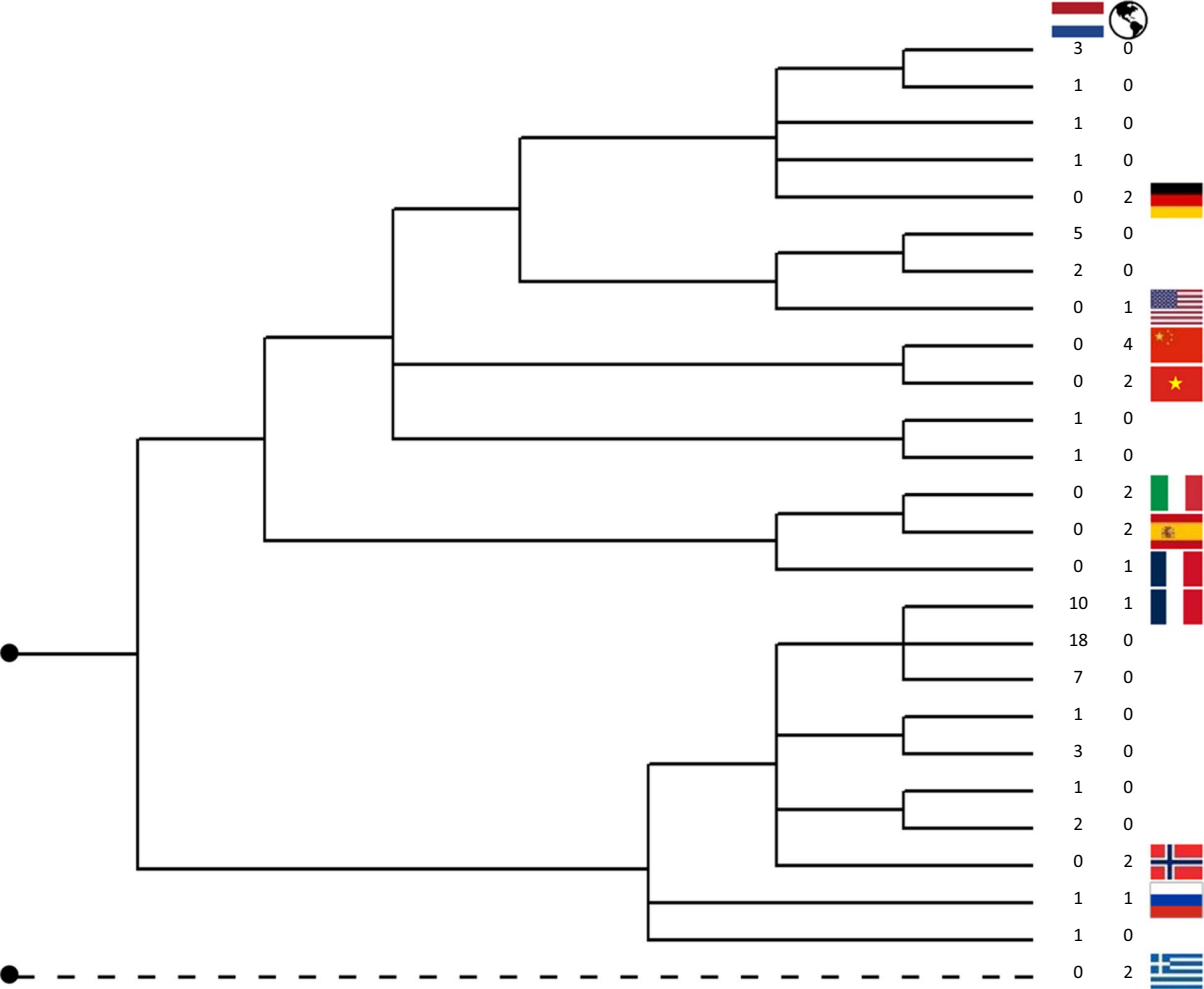
Polygenetic tree showing a globally shared *PLN* p.(Arg14del) variant haplotype, except for individuals from Greece (dashed line). The root node (black dot) of the phylogenetic tree represents a common ancestor. At the end of each branch, which represents a completely shared haplotype, the number of *PLN* p.(Arg14del)-positive carriers from the Netherlands and other countries was shown. Closely clustered terminals indicate genetically similar haplotypes, but recombination may have occurred (shown as internal nodes with more than two branches). The individuals from Greece (dashed line) share a different haplotype

Haplotype marker analysis was performed in all 13 newly identified individuals with PLN p.(Arg14del) except for those from Iceland. Furthermore, 32 additional Dutch PLN p.(Arg14del) index patients were haplotyped. The relationship between these and previously described haplotypes is visualized by a phylogenetic tree in Fig. 2 and is elaborated in Supplementary Table 3. Except for the Greek patient, all patients share at least part of the haplotype, suggesting that recombination events may have occurred. The haplotype of the Russian PLN p.(Arg14del)-positive carrier and one of the two French carriers was similar to the haplotype of at least one Dutch individual. The PLN p.(Arg14del)-positive carriers from China and Vietnam, as well as the individuals from France, Italy and Spain, closely cluster to each other, and in their turn again closely relate to Dutch PLN p.(Arg14del)-positive carriers. In addition, carriers from Germany and the U.S.A. also closely cluster with the Dutch PLN p.(Arg14del)-positive individuals. The age of the haplotype between Dutch and these other nationalities is estimated between 275 and 425 years old, assuming 25 years per generation.

Haplotypes were derived from WGS data for the Icelandic patients. WGS has been performed on 20 of these Icelandic carriers, and data reveal that none of these Icelandic carriers share any of the 23 prespecified SNPs surrounding the *PLN* gene with the 58 Dutch carriers. Further analyses revealed that estimated haplotypes in both Icelandic and Dutch patients overlap within the genomic region of approximately ± 200 kb, defined by the first flanking SNPs surrounding *PLN* c.40_42delAGA (Supplementary Figs. 2 and 3). The presence of a smaller, but overlapping haplotype in Icelandic patients could be explained by a common founder, likely originating more than 1000 years ago (based on the method described by Machado et al.[44]).

In conclusion, these finding suggest that most analyzed individuals share at least part of a common haplotype with the exception of the Greek patients.

## Discussion

To our knowledge, this study provides one of the most comprehensive overviews of the global prevalence and distribution of a single pathogenic variant underlying monogenic cardiac disease, i.e. PLN p.(Arg14del)-associated cardiomyopathy. This variant has so far been identified in 21 countries across four continents, with two additional countries awaiting confirmation. So far, over 2000 individuals have been identified globally, with most patients identified in the Netherlands. All tested patients, except those from Greece, share at least part of a common haplotype, suggesting a common ancestor who likely lived between 275 and 425 years ago (1600–1750 CE). This haplotype is thought to originate from the Dutch population, as previous estimations suggests that the haplotype within the Dutch PLN population emerged between 575 and 825 years ago [40]. The PLN variant in the Greek patients are most likely the result from an independent mutational event. The haplotype of the Icelandic patients is smaller (< 200 kb) than that observed in the Dutch population and could be either inherited through a distant ancestor (more than 1000 years ago) or could be caused by recombination.

The observed geographical distribution to different continents may reflect historical patterns of trade and migration. First, the calculated age of the common haplotype (275–425 years) aligns with the era when the Netherlands established extensive sea trade routes under the United Dutch East India Company (1602–1800 CE) [50]. The Dutch maintained a vast network of trading posts covering Africa and Asia (for an overview, see Supplementary Table 4. Strikingly, several of these trading posts correspond to countries where PLN p.(Arg14del) carriers have been identified, including China, India, Vietnam, and Japan. Furthermore, the Dutch were Japan’s sole trading partner for over two centuries and resided under severe restriction on the small artificial and gated island of Dejima. During this time, the generally male tradesmen were permitted to have relationships and establish families with Japanese women [51]. This could potentially have contributed to the observed distribution. In addition to trade with Asia, intensive trade occurred between the Northern and Southern European countries, i.e. under the Chamber of Levante Trade (1625–1826) [52]. This may explain the presence of PLN p.(Arg14del) carriers in countries like, Belgium, Germany, Norway, Russia, Sweden, Spain and Turkey.

Second, the variant may have spread through migration in more recent years. While emigration from the Netherlands to North America was present at relatively low levels during the seventeenth and eighteenth centuries, it increased significantly in the nineteenth and twentieth century (Canada, U.S.A., Brazil, Australia, South Africa) [5]. Emigration with a known Dutch ancestor occurred within PLN p.(Arg14del)-positive families in Australia, Canada, Italy[19], and U.S.A. (personal communications). However, no individuals with PLN p.(Arg14del) have been identified in Brazil and South Africa yet. In addition, van der Zwaag et al. described that the paternal ancestors of the PLN p.(Arg14del) patient from the U.S.A. were German/Norwegian [40]. In addition, our haplotype marker analysis confirms that the U.S.A. PLN haplotype is more closely related to the German PLN haplotype than to the Norwegian PLN haplotype (Fig. 2 and Supplementary Table 3). Finally, very recent emigration (< 75 years ago) of PLN p.(Arg14del)-positive individuals has occurred from the Netherlands to France, the UK and Spain as well as from Norway to Denmark (personal communication physicians).

Interestingly, the Greek family [7] has a different haplotype and therefore may represent an independent mutational event. The PLN variant c.40_42delAGA, a three base pairs deletion within the repeat unit “AAGAAG”, may suggest replication strand slippage as the underlying mechanism. During replication, repeat units can transiently dissociate and misalign, resulting in small insertions or deletions (indels) [53]. The likelihood of replication depends on the length and number of repeat units, and deletions occurring in the presence of only two repeat units, as in PLN, are considered rare [53, 54]. Furthermore, the chance of a de novo occurrence of PLN p.(Arg14del) is believed to be even less common*.* Germline de novo mutation rates for small indels have been estimated between 0.68 × 10^–9^ and 1.5 × 10^−9^ per base per generation [55, 56]. Given these very low occurrence rates, alternative explanations should be considered, including a sample mix-up, or the patients may carry a smaller haplotype, as has been observed in the Icelandic patients.

### Global relevance and future research

Since the initial description of PLN p.(Arg14del) in 2006, this variant has been identified in 21 countries across four continents, with data suggesting its presence in two additional countries. Its global prevalence, along with increasing availability of genetic testing, underscores the importance for geneticists and cardiologists worldwide to be aware of PLN p.(Arg14del)-associated cardiomyopathy. Because *PLN* was recently included in the ACMG’s (v3.3) list of recommended genes for reporting secondary findings [57], it is anticipated that more cases will be identified. Studying the global prevalence of PLN p.(Arg14del) and its haplotypes may provide the opportunity to explore factors underlying its clinical variability by examining patient cohorts from different geographic regions. Data on phenotypic expression in a more diverse study population facilitates further research on the effect of ethnic background on disease expression in PLN p.(Arg14del) cardiomyopathy. Additionally, to predict life-threatening ventricular arrhythmias and improve patient outcomes, a PLN p.(Arg14del) specific risk calculator was developed to identify those patients who may benefit from ICD implantation as primary prevention for sudden cardiac death [29, 30]. To increase reliability of the current risk model, additional patient cohorts with this specific variant will enable its external validation and provide the basis for risk prediction improvements.

Finally, novel promising precision medicine approaches targeting PLN p.(Arg14del) cardiomyopathy are emerging [31], such as gene editing, gene silencing, and signal transduction modulation. However, given the known variability in disease onset and severity in PLN p.(Arg14del) cardiomyopathy, identifying those who are most likely to benefit from these targeted therapies, and determining the optimal timing for intervention, remains challenging. Studies like ours contribute to a better overview of available patients across diverse populations for the study of natural history and potential disease modifiers that may help to further determine which patients would derive the greatest benefit from such promising treatment strategies.

### Strengths and limitations

This study substantially expands on previous work by examining the global prevalence and haplotypes of a single pathogenic gene variant associated with this increasingly recognized cardiomyopathy. Regional differences in the availability of genetic testing, combined with local and (inter)national varying extent of genetic testing panels, may have contributed to differences in the yield of PLN p.(Arg14del). Furthermore, even though we have explored various databases, the PLN p.(Arg14del) variant may not have been annotated in all sources leading to a potential underestimation of this variant.

## Conclusions

The PLN p.(Arg14del) variant underlying cardiomyopathy has been identified in at least 21 countries across four continents, with over 2000 carriers reported to date. While most carriers share at least part of a common haplotype suggesting common ancestry, our findings also suggest an additional independent mutational event in Greece. Given its observed distribution, it is likely that additional PLN p.(Arg14del) carriers will be identified in other countries, particularly those with historic connections to Europe, and more specifically, the Netherlands. Its global presence underscores the need for geneticists and cardiologists worldwide to be aware of PLN p.(Arg14del) cardiomyopathy in clinical practice. This study not only advances our understanding of its geographic and ancestral distribution, but also helps to identify patient cohorts that can support further research into factors underlying its clinical variability. Such efforts will be critical for optimizing patient selection for novel precision medicine initiatives including gene-based therapies.

## Supplementary Information

Below is the link to the electronic supplementary material.

Supplementary file1 (DOCX 24 KB)

Supplementary file2 (PPTX 952 KB)

Supplementary file3 (XLSX 21 KB)

## Data Availability

The datasets generated during the current study are available in the PLN_tracing respository, https://github.com/Jorisvansteenbrugge/PLN_Tracing.
