# Supplementary material for: Tracing the pathogenic PLN p.(Arg14del) variant across the globe; more than just a local curiosity
Source: J Cardiovasc Transl Res. 2026 Jun 25;19(1):78. doi: 10.1007/s12265-026-10792-6 (PMC13303328; doi:10.1007/s12265-026-10792-6)
Supplement: Supplementary file 1 — Supplementary file1 (DOCX 24 KB) [file 12265_2026_10792_MOESM1_ESM.docx]

**Supplementary Material**

Tracing the pathogenic PLN p.(Arg14del) variant across the globe; more than just a local curiosity

Esmée van Drie^1,2,3*^ Freyja H M van Lint^1,2,4*^, Rob Zwart^5^, Jie Wang^6^, Yucheng Chen^6^, Alex V Postma^5^, Martin G Elferink^1^, Joris J M van Steenbrugge^1^ Paul A van der Zwaag^7^, Jan DH Jongbloed^7^, Dennis Dooijes^1^, Myrthe Y C van der Heide^8^, Arjan C Houweling^5^, Kristina H Haugaa^9^, Ida Skrinde Leren^9^, Anna Kostareva^10^, Hendrik Milting^11^, Thuy Vy Nguyen^12^, Ho Huynh Thuy Duong^12,13^, Philippe Chevalier^3,14^, Antoine Delinière^14^, Juan R Gimeno-Blanes^3,15^, María Sabater^3,15^, Roberto Barriales-Villa^16^, Andrea Mazzanti^3,17^, Mirella Memmi^3,17^, Yuki Kuramoto^18^, Tomoka Tabata^18^, Arthur AM Wilde^3,8^, Karin Y van Spaendonck-Zwarts^1,2,7^, J Peter van Tintelen^1,3^

1. Department of Genetics, University Medical Center Utrecht, Heidelberglaan 100, 3584 CX Utrecht, the Netherlands
2. Netherlands Heart Institute, Moreelsepark 1, 3511 EP Utrecht, the Netherlands
3. Member of the European Reference Network for rare, low prevalence and complex diseases of the heart: ERN GUARD-Heart’ (ERN GUARDHEART; <http://guardheart.ern-net.eu>).
4. Department of Genetics, Radboud University Medical Center, Geert Grooteplein Zuid 10, 6525 GA Nijmegen, the Netherlands
5. Department of Human Genetics, Amsterdam UMC, Amsterdam, the Netherlands
6. Department of Cardiology, West China Hospital, Sichuan University, Guoxue Alley No. 37, Chengdu, 610041, Sichuan, China.
7. Department of Genetics, University of Groningen, University Medical Center Groningen, Hanzeplein 1, 9713 GZ Groningen, Netherlands
8. Amsterdam UMC location AMC, University of Amsterdam, Department of Cardiology, Meibergdreef 9, Amsterdam, the Netherlands; Amsterdam Cardiovascular Sciences, Heart Failure and arrhythmias, Amsterdam, the Netherlands
9. ProCardio center for research based innovation, Department of Cardiology, Oslo University Hospital, Rikshospitalet, Sognsvannsveien 20, Oslo 0372, Norway and University of Oslo, Oslo, Norway
10. Karolinska Institutet (KI) Solna, Sweden
11. Heart and Diabetes Center NRW, Bad Oeynhausen, Germany
12. Department of Genetics, Faculty of Biology and Biotechnology, University of Science, Vietnam National University, Ho Chi Minh City, Vietnam.
13. Research Center for Genetics and Reproductive Health, School of Medicine, Vietnam National University, Ho Chi Minh City, Vietnam.
14. Rhythmology Unit, Hospices Civils de Lyon, University of Lyon, Lyon, France.
15. Department of Cardiology, Virgen de Arrixaca Hospital, Ctra Murcia-Cartagena, s/n, 30120 El Palmar, Murcia, Spain
16. Inherited Cardiovascular Diseases Unit, Hospital Universitario A Coruña, As Xubias 84, INIBIC/ CIBERCV, 15006 A Coruña, Spain.
17. Department of Molecular Medicine, University of Pavia, Molecular Cardiology, ICS Maugeri, 27100, Pavia, Italy
18. Department of Cardiovascular Medicine, Osaka University Graduate School of Medicine, Japan

*Both authors contributed equally

Corresponding author: [e.vandrie@umcutrecht.nl](mailto:e.vandrie@umcutrecht.nl)

*Supplementary Note*

Identity-By-Descent (IBD) analysis:

If none of the 23 SNPs were shared, SNPs between the first two originally selected SNPs surrounding PLN c.40_42delAGA were selected (genomic interval of approximately +/- 200kb) and SNP phasing was performed with beagle v5.5 (1) at default settings using the 1000 Genomes Project bi-allelic SNP dataset as a reference panel. IBD between PLN p.(Arg14del) carriers from the Netherlands and from other countries was measured with PLINK v1.9 (2) using the ‘—cluster’ and ‘—mds-plot 3’ settings. SNPs in linkage disequilibrium were pruned before the IBD analysis with PLINK v1.9 using ‘—indep-pairwise’, a window size of 50, a step size of 5, and an R^2^ threshold of 0.4. Multidimensional scaling plots were visualized in R using ggplot2 (3). Subsequently, estimated haplotypes were compared between populations based on phased SNPs in the same region surrounding *PLN* c.40_42delAGA. A distance matrix between haplotypes was calculated using the manhattan distance, and haplotypes were clustered based with complete linkage using the ‘dist’ and ‘hclust’ methods in R respectively.

**References**

1. Browning BL, Tian X, Zhou Y, Browning SR. Fast two-stage phasing of large-scale sequence data. Am J Hum Genet. 2021;108(10):1880-90.

2. Chang CC, Chow CC, Tellier LC, Vattikuti S, Purcell SM, Lee JJ. Second-generation PLINK: rising to the challenge of larger and richer datasets. Gigascience. 2015;4:7.

3. Wickham H. ggplot2: Elegant Graphics for Data Analysis: Springer-Verlag New York; 2016.

*Supplementary Figures*

**Supplementary Figure 1 Schematic representation of haplotype analyses.**

Genomic positions of A) genetic markers, or B) 23 highly frequent single nucleotide polymorphisms (SNPs) surrounding the *PLN* p.(Arg14del) variant in whole genome sequencing data.

**Supplementary Figure 2 Multidimensional scaling plot demonstrating genetic clustering of Icelandic samples with Dutch PLN p.(Arg14del) individuals**

Panel A shows that Icelandic samples (green circles) cluster closely with Dutch PLN p.(Arg14del) individuals (blue circles) on the first two dimensions of genetic variation. Although in dimensions XX (panel B and C) Icelandic samples do not overlap with Dutch samples, four Dutch samples cluster more closely to Icelandic samples than other samples. Controls included one Dutch individual without the PLN p.(Arg14del) variant (purple circle) and a publicly available Northern European ancestry sample from Utah (red circle). Abbreviations: Dim: dimension.

**Supplementary Figure 3 Dendogram showing the genetic relationship between Icelandic and Dutch p.(Arg14del)-positive individuals**Each leaf corresponds to an Icelandic (“Carrier…”) or Dutch sample (“NL…”). Samples that are genetically related are combined into branches.
