# Supplementary material for: Tracing the pathogenic PLN p.(Arg14del) variant across the globe; more than just a local curiosity
Source: J Cardiovasc Transl Res. 2026 Jun 25;19(1):78. doi: 10.1007/s12265-026-10792-6 (PMC13303328; doi:10.1007/s12265-026-10792-6)
Supplement: Supplementary file 2 — Supplementary file2 (PPTX 952 KB) [file 12265_2026_10792_MOESM2_ESM.pptx]

## Slide 1
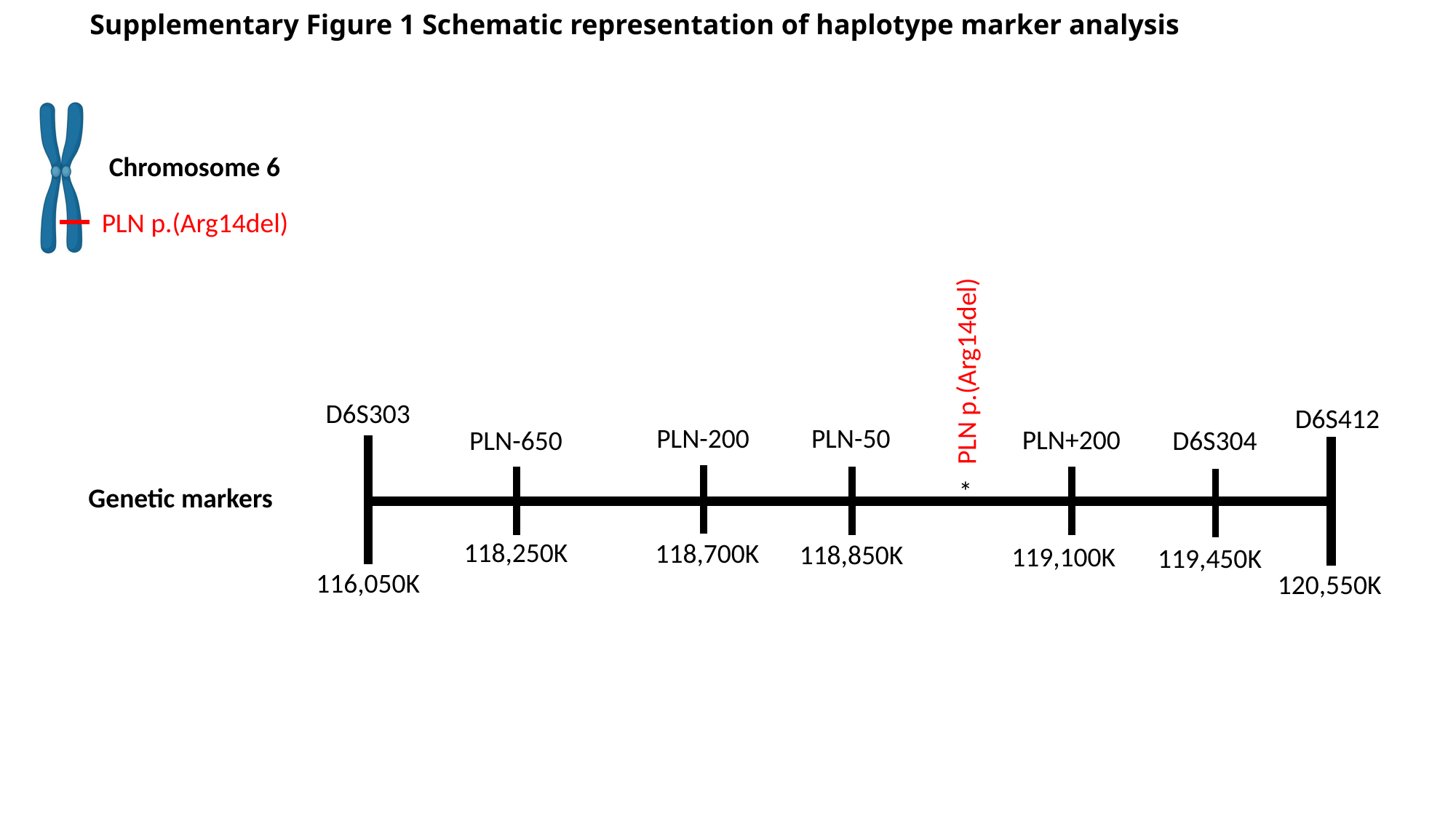

Supplementary Figure 1 Schematic representation of haplotype marker analysis
Chromosome 6
PLN p.(Arg14del)
PLN p.(Arg14del)
*
D6S303
D6S412
PLN-200
PLN-50
PLN+200
D6S304
PLN-650
Genetic markers
118,250K
118,700K
118,850K
119,100K
119,450K
116,050K
120,550K

## Slide 2
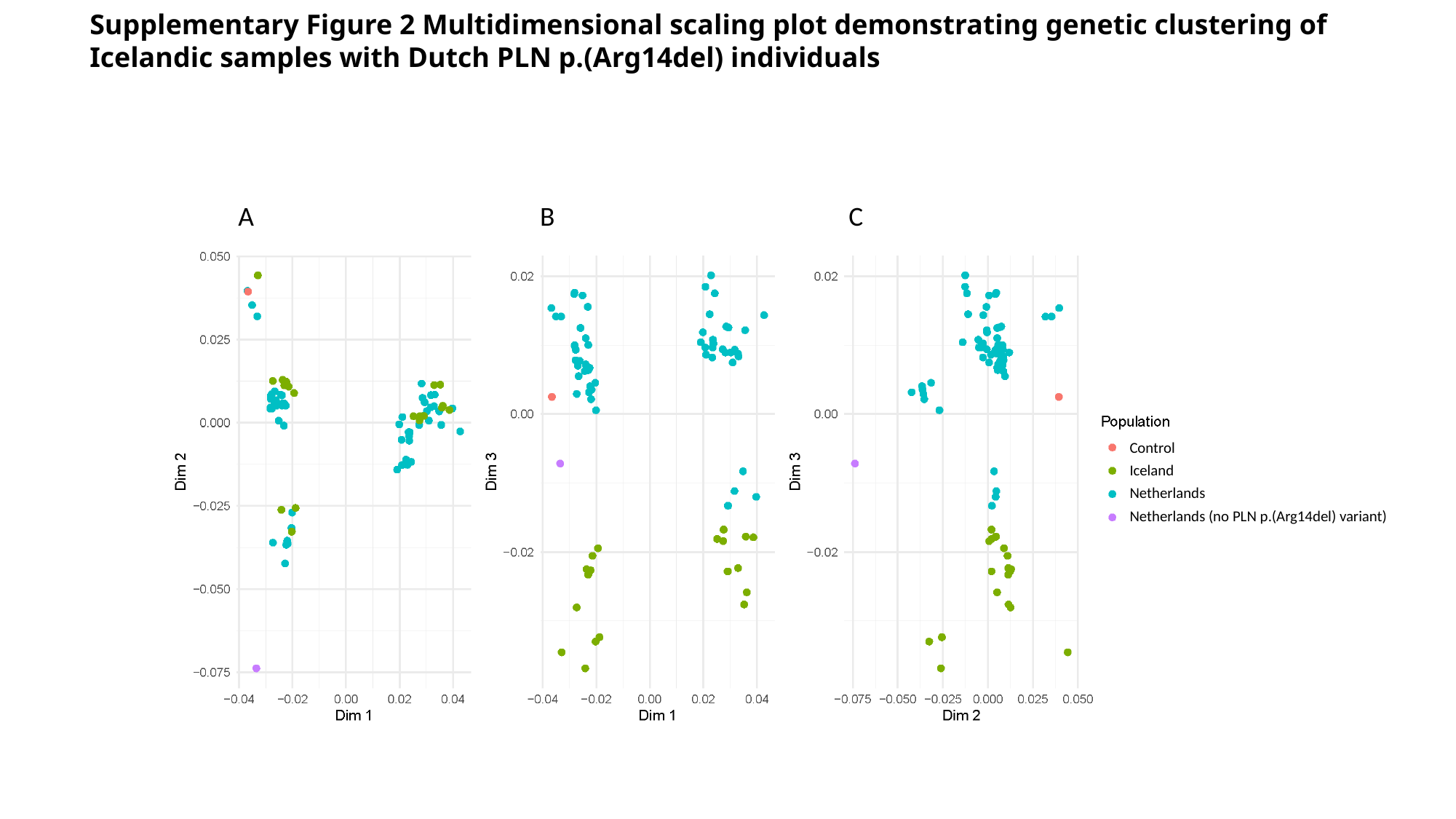

Supplementary Figure 2 Multidimensional scaling plot demonstrating genetic clustering of Icelandic samples with Dutch PLN p.(Arg14del) individuals
A
B
C
Control
Iceland
Netherlands
Netherlands (no PLN p.(Arg14del) variant)

## Slide 3
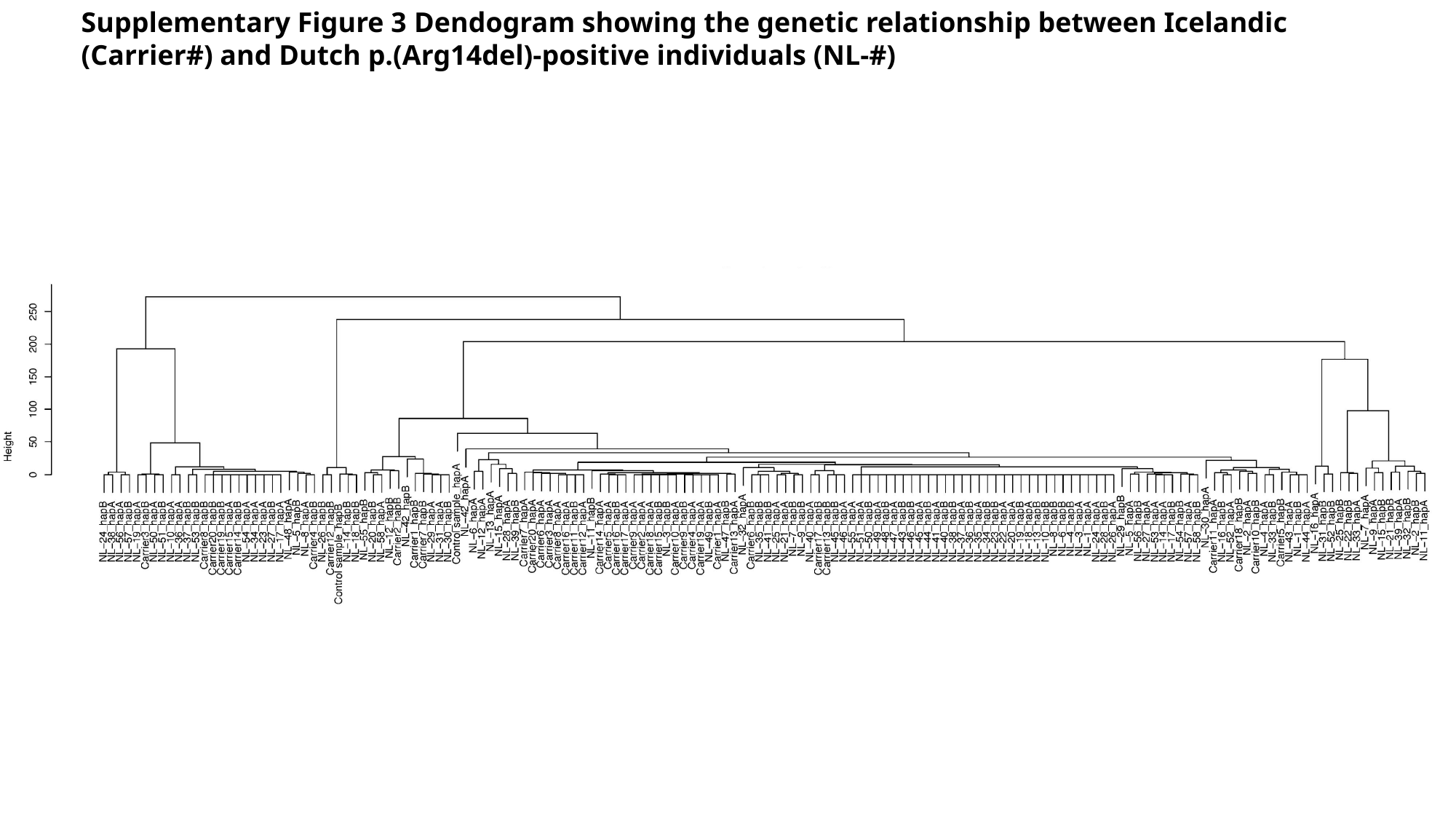

Supplementary Figure 3 Dendogram showing the genetic relationship between Icelandic (Carrier#) and Dutch p.(Arg14del)-positive individuals (NL-#)
